# Multi-omics for COVID-19: driving development of therapeutics and vaccines

**DOI:** 10.1093/nsr/nwad161

**Published:** 2023-05-30

**Authors:** Mengyu Guo, Muya Xiong, Jinying Peng, Tong Guan, Haixia Su, Yanyi Huang, Cai-Guang Yang, Yang Li, Diana Boraschi, Thanigaimalai Pillaiyar, Guanbo Wang, Chengqi Yi, Yechun Xu, Chunying Chen

**Affiliations:** CAS Key Laboratory of Biomedical Effects of Nanomaterials and Nanosafety, and CAS Center for Excellence in Nanoscience, National Center for Nanoscience and Technology of China, Beijing 100190, China; State Key Laboratory of Drug Research, Shanghai Institute of Materia Medica, Chinese Academy of Sciences, Shanghai 201203, China; University of Chinese Academy of Sciences, Beijing 100049, China; State Key Laboratory of Protein and Plant Gene Research, School of Life Sciences, Peking University, Beijing 100871, China; CAS Key Laboratory of Biomedical Effects of Nanomaterials and Nanosafety, and CAS Center for Excellence in Nanoscience, National Center for Nanoscience and Technology of China, Beijing 100190, China; University of Chinese Academy of Sciences, Beijing 100049, China; State Key Laboratory of Drug Research, Shanghai Institute of Materia Medica, Chinese Academy of Sciences, Shanghai 201203, China; University of Chinese Academy of Sciences, Beijing 100049, China; Biomedical Pioneering Innovation Centre, Peking University, Beijing 100871, China; Institute for Cell Analysis, Shenzhen Bay Laboratory, Shenzhen 528107, China; Peking-Tsinghua Center for Life Sciences, Peking University, Beijing 100871, China; College of Chemistry and Molecular Engineering, Beijing National Laboratory for Molecular Sciences, Peking University, Beijing 100871, China; State Key Laboratory of Drug Research, Centre for Chemical Biology, Shanghai Institute of Materia Medica, Chinese Academy of Sciences, Shanghai 201203, China; University of Chinese Academy of Sciences, Beijing 100049, China; Laboratory of Immunology and Nanomedicine, and China-Italy Joint Laboratory of Pharmacobiotechnology for Medical Immunomodulation, Shenzhen Institute of Advanced Technology, Chinese Academy of Sciences, Shenzhen 518055, China; Laboratory of Immunology and Nanomedicine, and China-Italy Joint Laboratory of Pharmacobiotechnology for Medical Immunomodulation, Shenzhen Institute of Advanced Technology, Chinese Academy of Sciences, Shenzhen 518055, China; Institute of Biochemistry and Cell Biology, National Research Council, Napoli 80131, Italy; Institute of Pharmacy, Pharmaceutical/Medicinal Chemistry and Tuebingen Center for Academic Drug Discovery, Eberhard Karls University Tübingen, Tübingen 72076, Germany; Biomedical Pioneering Innovation Centre, Peking University, Beijing 100871, China; Institute for Cell Analysis, Shenzhen Bay Laboratory, Shenzhen 528107, China; State Key Laboratory of Protein and Plant Gene Research, School of Life Sciences, Peking University, Beijing 100871, China; Department of Chemical Biology and Synthetic and Functional Biomolecules Center, College of Chemistry and Molecular Engineering, Peking University, Beijing 100871, China; Peking-Tsinghua Center for Life Sciences, Peking University, Beijing 100871, China; State Key Laboratory of Drug Research, Shanghai Institute of Materia Medica, Chinese Academy of Sciences, Shanghai 201203, China; University of Chinese Academy of Sciences, Beijing 100049, China; CAS Key Laboratory of Biomedical Effects of Nanomaterials and Nanosafety, and CAS Center for Excellence in Nanoscience, National Center for Nanoscience and Technology of China, Beijing 100190, China; University of Chinese Academy of Sciences, Beijing 100049, China; GBA National Institute for Nanotechnology Innovation, Guangzhou 510700, China

**Keywords:** COVID-19, SARS-CoV-2, multi-omics, antibodies, small-molecule drugs, vaccines

## Abstract

The ongoing COVID-19 pandemic caused by SARS-CoV-2 has raised global concern for public health and economy. The development of therapeutics and vaccines to combat this virus is continuously progressing. Multi-omics approaches, including genomics, transcriptomics, proteomics, metabolomics, epigenomics and metallomics, have helped understand the structural and molecular features of the virus, thereby assisting in the design of potential therapeutics and accelerating vaccine development for COVID-19. Here, we provide an up-to-date overview of the latest applications of multi-omics technologies in strategies addressing COVID-19, in order to provide suggestions towards the development of highly effective knowledge-based therapeutics and vaccines.

## Teaser

Understanding the roles of multi-omics in COVID-19 facilitates the development of novel therapeutics and provides guidance for the rational design of vaccines.

## INTRODUCTION

**Figure 1.**
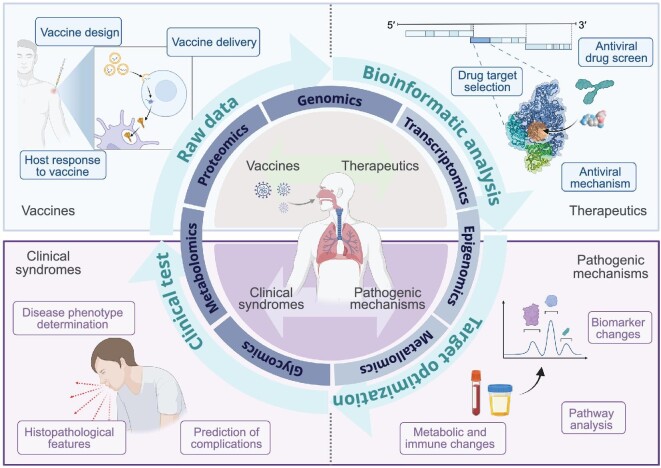
Multi-omics approaches facilitate drug and vaccine development. Created by Biorender.com.

With the development of high-throughput sequencing, mass spectrometry, and computer science and algorithms, various omics approaches, such as genomics, transcriptomics, epigenomics, proteomics and metabolomics, are now widely accessible, enabling the systematic and comprehensive analysis of life processes. The utilization of multi-omics data is expected to accelerate the development of new drugs and play an important role in vaccine design (Fig. 1). Typically, the discovery of new drugs, such as antibodies (Abs) and small molecules, is a lengthy process that relies on the identification of targets and the design and development of strategies to inhibit or activate them. Multi-omics strategies can be exploited to systematically study the pathogenesis of diseases, identify treatable targets, predict drug resistance, etc., thus greatly accelerating the progress of drug development.

Vaccination is the most effective way to prevent and control infectious diseases. The keys to vaccine development are the accurate and rapid identification of antigens specific to the pathogen, and the ability to induce a protective immune response with long-term immune memory. In recent years, proteomics and genomics advancements have made it possible to isolate and identify pathogen-associated antigens, thus increasing the effectiveness of vaccine antigen selection and accelerating vaccine development. Studies in genomics, proteomics and metabolomics have also enabled thorough investigation of host responses after vaccination, guiding the design of effective and safe vaccines.

In early 2020, a novel coronavirus, SARS-CoV-2, began to spread rapidly in humans, causing enormous health, economic and social impacts worldwide. The aforementioned multi-omics technologies played crucial roles in the study of SARS-CoV-2 and the related disease, COVID-19. In December 2020, researchers extracted serial plasma samples from 139 patients at various stages of COVID-19, quantified plasma proteins and metabolites, and sequenced peripheral blood mononuclear cell (PBMC) transcripts and surface proteins [1]. This integrated analysis provided useful information for therapeutic intervention. Another team used large-scale single-cell transcriptome profiling to reveal the immune signature of COVID-19 [2]. A single-cell RNA sequencing (RNA-seq) analysis was performed on 284 samples from 196 COVID-19 patients to build a comprehensive immune landscape from 1.46 million cells. Using the single-cell sequencing data, the authors identified changes in different circulating leukocyte subpopulations and patients’ characteristics, such as severity and stage of disease, in COVID-19 pneumonia. The authors identified COVID-19-associated RNA in multiple epithelial and immune cell types, accompanied by significant changes in the host cell transcriptome. Based on the increased understanding of SARS-CoV-2 and of the host responses to the virus, subsequent studies using multi-omics approaches yielded 84 potentially active compounds, and further computational and toxicity analyses led to the identification of six candidate drugs: amsacrine, bosutinib, cretinoin, crizotinib, nidanib and sunitinib [3]. In recent years, metallomics has also gradually stepped into a new era and has been integrated with other disciplines. In response to the current SARS-CoV-2 pandemic, some scholars have proposed using a comparative metallomics approach to screen COVID-19 drugs. An anti-ulcer drug already in use, bismuth ranitidine citrate, was identified as potentially active against SARS-CoV-2 by a metallomics approach and is expected to be applied for the treatment of COVID-19 [4].

Multi-omics technologies have helped uncover the molecular processes and host responses underlying COVID-19 initiation, progression and transmission. In this review, we present an overview of how multi-omics approaches have aided both our understanding of the pathogenesis of COVID-19 pneumonia and the development of effective therapeutic options (Abs and small-molecule drugs) and vaccines. The application of multi-omics to COVID-19 has accelerated our ability to develop novel therapies and offers a direction for the logical design of future vaccines.

### Design of antibodies against SARS-CoV-2

Abs, produced by plasma cells and specifically targeting antigens, have become the predominant treatment modality for various diseases over the past decades due to their high specificity and favorable pharmacokinetic properties. Despite the generally long development cycle for Ab therapeutics, the discovery and development of Abs for the prevention and treatment of COVID-19 were conducted in an expedited manner in response to the pandemic.

#### Identification of neutralizing antibodies against wild-type SARS-CoV-2

Although neutralizing antibodies (NAbs) in convalescent plasma from patients have induced clinical improvement in mild and severe COVID-19 patients [5], the therapeutic use of such NAbs is limited due to insufficient scalability. Isolating monoclonal antibodies (mAbs) with neutralizing capability from convalescent patients’ memory B cells provided a promising solution for intervention against the SARS-CoV-2 infection. The diverse B cell repertoires generated by VDJ (variable, diversity and joining) recombination and somatic hypermutation require mAb sequence information to be obtained from clonal amplification of B cells. NAbs for SARS-CoV-2 can be isolated using single-cell RNA and VDJ sequencing of antigen-specific memory B cells from individuals who recovered from a SARS-CoV-2 infection, individuals who had been vaccinated against SARS-CoV-2, and SARS-CoV-1 convalescent individuals [6]. To circumvent the requirement for biosafety level 3 (BSL-3) conditions when handling the highly pathogenic and infectious live SARS-CoV-2, which hinders the development of therapeutics, pseudovirus-based neutralization assays were developed for evaluating NAbs against SARS-CoV-2 in biosafety level 2 (BSL-2) facilities [7]. Within a few months after the first reported outbreak of COVID-19, several groups identified potent NAbs that were anticipated to be candidates for the development of clinical intervention against SARS-CoV-2. Pinto *et al.* identified the S309 mAb, which cross-reacts with the S glycoprotein of SARS-CoV-2 and potently neutralized the virus, isolated from memory B cells of an individual infected with SARS-CoV-1 in 2003 [8]. Wu *et al.* isolated four NAbs from a convalescent patient, two of which showed complete competition with ACE2 (angiotensin‐converting enzyme 2) for binding to the receptor binding domain (RBD) of the SARS-CoV-2 S glycoprotein [9]. Additionally, 14 potent SARS-CoV-2 NAbs were identified from 8558 antigen-binding IgG1^+^ clonotypes from 60 COVID-19 convalescent patients. The most potent of these NAbs exhibited strong therapeutic and prophylactic efficacy in a SARS-CoV-2-infected mild-symptom mouse model and a severe-symptom hamster model [10,11]. From 8 individuals infected with SARS-CoV-2, 206 RBD-specific NAbs were isolated and characterized from single B cells; these NAbs exhibited a wide range of RBD-binding activities [12]. Through single-cell sorting of S-glycoprotein-specific memory B cells from COVID-19 convalescent individuals, 453 NAbs were identified, the most potent of which could neutralize the virus at very low doses, exhibiting significant prophylactic and therapeutic efficacy in a hamster COVID-19 model [13]. Furthermore, two potent NAbs were isolated from a convalescent patient; the most potent of these demonstrated therapeutic potential through inhibition of infection in rhesus monkeys in both prophylactic and therapeutic settings [14]. Table 1 summarizes the representative NAbs for the wild-type SARS-CoV-2 identified and characterized in these studies.

**Table 1.** Potent NAbs for the wild-type SARS-CoV-2 identified and characterized in representative investigations.

Number of mAbs isolated or characterized	Number of NAbs characterized^a^	Origin	Most potent NAb	Structural characterization	Target (conformation, if applicable)	Epitope	Ref.
15	4	1 convalescent COVID-19 patient	B38	X-ray crystallography	RBD	36 residues in segments 403–505	[9]
			H4			N/A	
169	14	60 convalescent COVID-19 patients	BD-368–2				[10]
			BD-368–2	Cryo-EM	RBD (up and down)	Including ACE2 binding sites	[11]
25	1	1 individual infected with SARS-CoV	S309	Cryo-EM	BD (up and down)	Residues 337–344 of SB, and a glycan at position N343	[8]
206	3	8 individuals infected with SARS-CoV-2	P2C-1F11	X-ray crystallography	N/A	N/A	[12]
			P2B-2F6		RBD (up and down)	Receptor-binding motif, including K444, G446, G447, N448, Y449, N450, L452, V483, E484, G485, F490 and S494	
			P2C-1A3		N/A	N/A	
N/A	2(1)	1 patient convalescing from COVID-19	CB6	X-ray crystallography	RBD	Largely overlap with the binding epitopes of ACE2	[14]
N/A	1	Immunized mice	47D11	N/A	RBD	N/A	[167]
45	2	1 patient infected with SARS-CoV-2	CV30	N/A	RBD	N/A	[168]
>1000	53	17 SARS-CoV-2–recovered individuals	CC6.29 and CC6.30	N/A	RBD	N/A	[169]
84	19(**7**)	3 individuals infected with SARS-CoV-2	COVA1-18	N/A	RBD	N/A	[170]
			COVA2-15	Negative-stain EM	RBD (up and down)	Partially overlapped with the ACE2-binding site	
89	52	149 convalescent COVID-19 individuals	C121 and C002	Negative-stain EM	RBD (up and down)	N/A	[171]
10^3^ level	>200	Immunized mice; and 3 convalescent COVID-19 individuals	REGN10987	Hydrogen/deuterium exchange mass spectrometry and cryo-EM	BD	Side of RBD, with little to no overlap with the ACE2 binding site	[172]
399	3	10 convalescent COVID-19 individuals	4A8	Cryo-EM	TD	Residues 141 to 156, and 246 to 260	[15]
252	19(9)	5 patients infected with SARS-CoV-2	2–15, 2–7, 1–57, and 1–20	ELISA-based epitope mapping	RBD	N/A	[31]
			2–17 and 5–24		NTD		
			4–8	Cryo-EM			
			2–43	Cryo-EM	Undetermined regions on spike	A quaternary epitope on the top of the spike that included elements of the RBDs from two adjacent S1 protomers	
			2–51	ELISA-based epitope mapping		N/A	
389	95	2 convalescing COVID-19 individuals					[173]
	40	2 convalescing COVID-19 individuals	COV2-2196	Negative-stain EM	RBD (up)	ACE2 recognition motif	[174]
N/A	N/A	Mice immunized with recombinant SARS-CoV RBD	H014	Cryo-EM	BD (up and down)	A cavity on one side of the RBD comprising residues 368–386, 405–408, 411–413, 439 and 503	[175]
		2 individuals recovering from severe COVID-19	S2E12	Cryo-EM	BD (up)	An epitope overlapping with the receptor-binding motif (RBM), including residues 455 to 458 and 473 to 493	
			S2M11	Cryo-EM	RBD (down)	A quaternary epitope, including the RBM crevice; the interface between the RBM and helices 339 to 343 and 367 to 374; residue 436 of an adjacent RBD	[176]
598	40 (18)	10 COVID-19 patients	CV07-209	N/A	RBD	N/A	[177]
			CV07-250	X-ray crystallography		Completely overlaps with the ACE2 binding site with a similar angle of approach as ACE2	
377	20	42 individuals infected with SARS-CoV-2	384	X-ray crystallography and cryo-EM	RBD (up and down)	482–486 on top of the shoulder	[35]
92	27	14 individuals recovered from COVID-19	BG10-19	Cryo-EM	RBD (down)	Quaternary epitope comprising interactions that bridge two neighboring RBDs and the N165_NTD_-glycan on the adjacent NTD	[178]

a The numbers in bracket represent potent NAbs defined in the corresponding reports.

The main target of NAbs with regard to coronavirus infection is the spike protein (S), because it is surface-exposed and mediates cell entry by binding to ACE2 on target cells [15]. Analysis of the rich proteomic and genomic data in the international immunogenetics information system (IMGT) database revealed that the natural, all-human combinatorial antibody library constructed 20 years ago contains NAbs against the S glycoprotein of SARS-CoV-2 [10]. That study also revealed that the combinatorial antibody library, like the human immune system *in vivo*, can generate effective recognition sequences in response to antigenic stimulation. These findings provide new directions for the future development of Abs against SARS-CoV-2, as well as vaccines. While most NAbs isolated from COVID-19 convalescent patients target the RBD, some NAbs recognize the N-terminal domain (NTD) of the S glycoprotein [16]. The NTD-directed NAbs are generally less potent, probably because of their inability to compete with ACE2 binding [17]. It is noteworthy that the Ab-spike binding affinities do not fully correlate with the neutralizing abilities, because Ab-spike binding does not necessarily ensure blocking the spike-ACE2 binding [16]. For example, as an NAb targeting the RBD of SARS-CoV, CR3022 can bind to the RBD of SARS-CoV-2 with high affinity as well, but it is unable to neutralize SARS-CoV-2 [18]. Therefore, binding-based screening of spike-targeting Abs is often followed by function-based screening and epitope mapping during identification of potent NAbs.

#### Identification of neutralizing antibodies against SARS-CoV-2 variants

**Figure 2.**
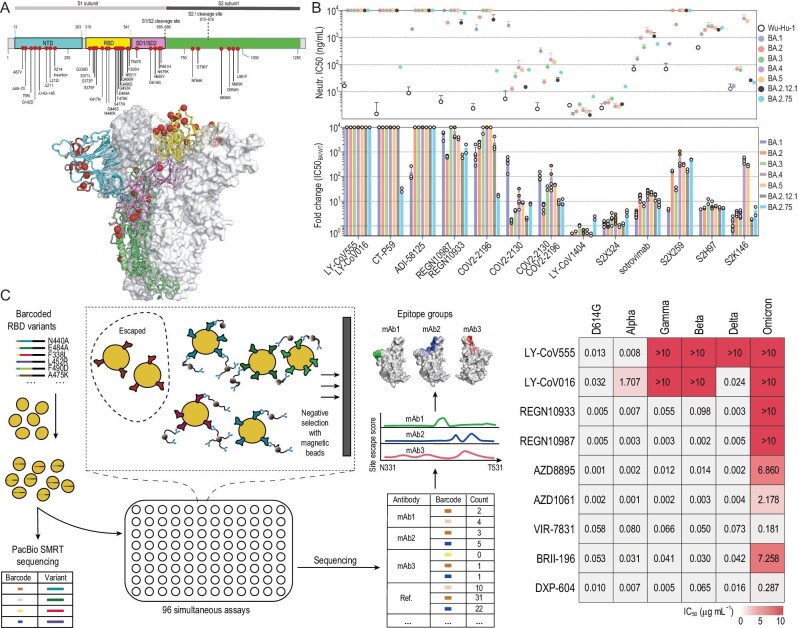
Screening of NAbs that avoid Omicron escape. (A) The structure of the BA.1 (Omicron) variant of the SARS-CoV-2 spike trimer. The NTD, RBD, subdomains 1 and 2, and the S2 protein are shown in cyan, yellow, pink and green, respectively. The red spheres indicate the alpha carbon positions for each Omicron variant residue. NTD-specific loop insertions/deletions are shown in red, with the original loop shown in transparent black. Adapted with permission from [30]. (B) mAb-mediated neutralization of vesicular stomatitis virus (VSV) pseudoviruses carrying the mutations present in the S glycoprotein of the Omicron variants BA.1, BA.2, BA.3, BA.4, BA.5, BA.2.12.1 and BA.2.75. The potency of each mAb or mAb cocktail is indicated by their IC_50_ (top) or fold change relative to neutralization of the Wuhan-Hu-1 (D614) pseudovirus (bottom). Adapted with permission from [25]. (C) Schematic of MACS-based high-throughput yeast display mutation screening (left), and neutralization of SARS-CoV-2 VOCs (pseudotyped VSV) by nine NAbs (right). Adapted with permission from [6].

As an RNA virus, SARS-CoV-2 evolves continuously via the occurrence of genetic mutations or viral recombination during replication. Numerous mutations have been revealed through genetic sequencing. The selective pressure from Abs induced by SARS-CoV-2 infection may also promote additional mutations [19,20]. Although most mutations are neutral or mildly deleterious, a small portion of mutations are fitness enhancing and may alter various aspects of virus biology, such as pathogenicity, infectivity, transmissibility and antigenicity [21,22]. Table 2 summarizes the mutations of interest in the S glycoproteins that have been detected up to 22 September 2022. For example, the variant B.1.1.529 (designated Omicron) contains more than 30 mutations in the S glycoprotein (Fig. 2A). Because essentially all NAbs found in the sera of convalescent individuals or elicited by vaccines target epitopes in the S glycoprotein of SARS-CoV-2 (RBD, NTD, or other epitopes), a mutation in S, an event that contributes to the spreading of SARS-CoV-2 variants, especially global variants of concern (VOCs), can cause escape from NAbs [23]. VOCs show decreased susceptibility to previously identified NAbs raised against wild-type SARS-CoV-2 or its other variants (Fig. 2B) [24,25]. The effect of SARS-CoV-2 mutations on viral antigenicity can be revealed by testing the ability of NAbs to bind either isolated mutants or pseudotyped viruses that carry the mutations [24,25]. Mutation screening using a fluorescence-activated cell sorting (FACS)-based yeast display platform not only allows mapping of all single-amino-acid mutations that affect the binding of specific NAbs but also predicts the efficacy of NAb-based drugs against mutations [26–29]. To assess how a large collection of NAbs reacts to SARS-CoV-2 variants, screening against mutations can be performed by magnetic-activated cell sorting (MACS), which increases throughput ∼100-fold over FACS while maintaining comparable data quality [6] (Fig. 2C).

**Table 2.** Key mutations in SARS-CoV-2 variants (collected from the European Centre for Disease Prevention and Control, ECDC^50^).

WHO	Lineage and	Country			
label	additional mutations	first detected	Spike mutations of interest	First detected	Designated VOC
Alpha	B.1.1.7	United Kingdom	N501Y, D614G, P681H	Sep-20	Dec-20
Beta	B.1.351	South Africa	K417N, E484K, N501Y, D614G, A701V	Sep-20	Jan-21
Epsilon	B.1.427/B.1.429	USA	L452R, D614G	Sep-20	
n/a	C.16	Unclear	L452R, D614G	Oct-20	
n/a	B.1.526.1	USA	L452R, D614G	Oct-20	
n/a	B.1.1.519	Mexico	T478K, D614G	Nov-20	
n/a	B.1.1.7 + E484K	United Kingdom	E484K, N501Y, D614G, P681H	Dec-20	
Eta	B.1.525	Nigeria	E484K, D614G, Q677H	Dec-20	
Kappa	B.1.617.1	India	L452R, E484Q, D614G, P681R	Dec-20	
n/a	B.1.214.2	Unclear	Q414K, N450K, ins214TDR, D614G	Dec-20	
n/a	A.23.1 + E484K	United Kingdom	V367F, E484K, Q613H	Dec-20	
n/a	A.27	Unclear	L452R, N501Y, A653V, H655Y	Dec-20	
n/a	A.28	Unclear	E484K, N501T, H655Y	Dec-20	
n/a	B.1.351 + P384L	South Africa	P384L, K417N, E484K, N501Y, D614G, A701V	Dec-20	
Iota	B.1.526	USA	E484K, D614G, A701V	Dec-20	
n/a	B.1.526.2	USA	S477N, D614G	Dec-20	
n/a	C.36 + L452R	Egypt	L452R, D614G, Q677H	Dec-20	
Lambda	C.37	Peru	L452Q, F490S, D614G	Dec-20	
Gamma	P.1	Brazil	K417T, E484K, N501Y, D614G, H655Y	Dec-20	Jan-21
Delta	B.1.617.2	India	L452R, T478K, D614G, P681R	Dec-20	May-21
Theta	P.3	The Philippines	E484K, N501Y, D614G, P681H	Jan-21	
n/a	B.1.351 + E516Q	Unclear	K417N, E484K, N501Y, E516Q, D614G, A701V	Jan-21	
n/a	B.1.1.7 + L452R	United Kingdom	L452R, N501Y, D614G, P681H	Jan-21	
n/a	B.1.1.7 + S494P	United Kingdom	S494P, N501Y, D614G, P681H	Jan-21	
Zeta	P.2	Brazil	E484K, D614G	Jan-21	
n/a	AT.1	Russian Federation	E484K, D614G, N679K, ins679GIAL	Jan-21	
Mu	B.1.621	Colombia	R346K, E484K, N501Y, D614G, P681H	Jan-21	
n/a	B.1.1.318	Unclear	E484K, D614G, P681H	Jan-21	
n/a	B.1.616	France	V483A, D614G, H655Y, G669S	Feb-21	
n/a	B.1.620	Unclear	S477N, E484K, D614G, P681H	Feb-21	
n/a	B.1.617.3	India	L452R, E484Q, D614G, P681R	Feb-21	
n/a	P.1 + P681H	Italy	D614G, E484K, H655Y, K417T, N501Y, P681H	Feb-21	
n/a	AV.1	United Kingdom	N439K, E484K, D614G, P681H	Mar-21	
n/a	B.1.617.2 + E484X	India	L452R, T478K, D614G, P681R, E484X	Apr-21	
n/a	B.1.617.2 + Q613H	India	L452R, T478K, D614G, P681R, Q613H	Apr-21	
n/a	B.1.617.2 + Q677H	India	L452R, T478K, D614G, P681R, Q677H	Apr-21	
n/a	AY.4.2	United Kingdom	L452R, T478K, D614G, P681R, A222V, Y145H	Jun-21	
n/a	B.1.617.2+K417N	United Kingdom	L452R, T478K, D614G, P681R, K417N	Jun-21	
n/a	C.1.2	South Africa	D614G, E484K, H655Y, N501Y, N679K, Y449H	Jun-21	
n/a	B.1.640	The Republic of Congo	D614G, F490R, N394S, N501Y, P681H, R346S, Y449N, 137−-145de	Sep-21	
Omicron	BA.1	South Africa and Botswana	A67V, Δ69-70, T95I, G142D, Δ143-145, N211I, Δ212, ins215EPE, G339D, S371L, S373P, S375F, K417N, N440K, G446S, S477N, T478K, E484A, Q493R, G496S, Q498R, N501Y, Y505H, T547K, D614G, H655Y, N679K, P681H, N764K, D796Y, N856K, Q954H, N969K, L981F	Nov-21	Nov-21
Omicron	BA.2	South Africa	G142D, N211I, Δ212, V213G, G339D, S371F, S373P, S375F, T376A, D405N, R408S, K417N, N440K, S477N, T478K, E484A, Q493R, Q498R, N501Y, Y505H, D614G, H655Y, N679K, P681H, N764K, D796Y, Q954H, N969K	Nov-21	
Omicron	BA.3	South Africa	A67V, Δ69-70, Δ143-145, N211I, Δ212, G339D, S371F, S373P, S375F, D405N, K417N, N440K, G446S, S477N, T478K, E484A, Q493R, Q498R, N501Y, Y505H, D614G, H655Y, N679K, P681H, D796Y, Q954H, N969K	Nov-21	
n/a	XF	United Kingdom	Omicron-like	Jan-22	
n/a	XD	France	NTD Delta-like; remaining Omicron-like	Jan-22	
Omicron	BA.4	South Africa	L452R, F486V, R493Q	Jan-22	
Omicron	BA.5	South Africa	L452R, F486V, R493Q	Feb-22	
Omicron	BA.2.75	India	W152R, F157L, I210V, G257S, D339H, G446S, N460K, Q493 (reversion)	May-22	
Omicron	XAK	Germany	K147E, N460K, G339D, Δ69	Jun-22	
Omicron	BA.2+L452X	n/a	L452X	n/a	
Omicron	BF.7	United Kingdom	L452R, F486V, R493Q, R346T	July-02	

**Figure 3.**
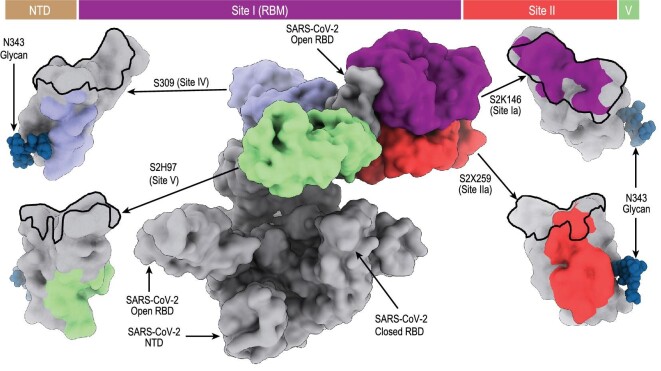
The RBD sites targeted by four mAbs that cross-neutralize Omicron. Representative Abs (the Fv region) bound to spike proteins are shown as a composite. Colored surfaces on the RBD depict the epitopes, and the RBM is shown as a black outline. Adapted from [36].

To identify mAbs reactive to SARS-CoV-2 variants, researchers have engaged in the identification and development of broadly neutralizing mAbs, which can have a wider therapeutic efficacy [31]. Viable candidates for prophylactic or therapeutic protection against a broad range of SARS-CoV-2 variants were identified from either recovered patients or humanized mice [32–34]. An *in vitro* evolution approach using random mutagenesis and selection by yeast display libraries enabled the generation of engineered NAbs with broad and potent antiviral activity against SARS-related viruses [35]. The Beijing government has approved the use of DXP-604 (NCT04669262 and NCT05381519), a potent, broad-spectrum NAb identified by Cao *et al.* [7] (Fig. 2C), as a compassionate drug in Beijing Ditan Hospital. Thirty-five patients have received DXP-604; as of 19 November 2022, 17 of the patients had recovered successfully. Cameroni *et al.* compared the *in vitro* neutralizing activity of 36 neutralizing NTD- or RBD-specific mAbs against the Omicron (B.1.1.529) variant. The NTD targeting mAbs lost all activity against B.1.1.529, whereas four classes of mAbs, defined by their cognate RBD-binding sites (sites I, II, IV and V), retained activity: S2K146, S2×324 and S2N28 targeting site I (RBM), S2×259 targeting conserved RBD site II, sotrovimab targeting site IV, and S2H97 targeting highly conserved cryptic site V (Fig. 3) [36].

Combinations of NAbs with distinct, non-overlapping epitopes can provide greater protection and resistance against mutation than single mAbs [37]. Such NAbs can be selected from the previously identified panels of NAbs for the wild-type or earlier variants of SARS-CoV-2 [38]. NTD-targeting mAbs were suggested for use in cocktail therapies with RBD-targeting mAbs [39,40]. Treatment with a cocktail that includes Abs that recognize different non-overlapping epitopes of the RBD was shown to minimize the generation of escape mutations [19]. Differentiation among antibody epitopes through high-resolution structural characterization facilitated the development of Abs with greater resistance to immune escape [34]. CoV-AbDab, a public database released by the Oxford Protein Informatics Group, documents all published or patented Abs and nanobodies able to bind to SARS-CoV-2 and other coronaviruses, including SARS-CoV-1 and MERS-CoV [41]. As of 26 July 2022, there have been 8802 entries annotated as SARS-CoV-2-binding deposited in CoV-AbDab.

Multiple anti-SARS-CoV-2 Ab products, developed as either monotherapies or a combination, have either requested authorization or been granted approval by the US FDA, although these authorizations will terminate at the end of the pandemic [42]. With the circulation of new VOCs, the use of certain mAbs can be limited by the US FDA if these treatments are shown to be inactive against the variants. As an example, the US FDA issued emergency use authorization (EUA) for bamlanivimab, administered as a monotherapy, in November 2020. But in April 2021, due to the increased resistance of VOCs to the mAb, the EUA was revoked. However, bamlanivimab and etesevimab administered together received an EUA in February 2021 for the treatment of mild-to-moderate COVID-19 in adults and pediatric patients who test positive for SARS-CoV-2 and who are at high risk of progressing to severe COVID-19. The emergency use of these Abs as postexposure prophylaxis for COVID-19 in adults and pediatric patients (12 years of age and older, weighing at least 40 kg) at high risk of progression to severe COVID-19 was later included in the revised EUA. In December 2021, the National Medical Products Administration (NMPA) of China approved the registration of applications for the NAb combination therapy drugs ambavir monoclonal injection (BRII-196) and romesvir monoclonal injection (BRII-198) [12], the first time independent intellectual property rights had been awarded to a SARS-CoV-2 NAb combination drug in China.

#### Identification of nanobodies against SARS-CoV-2

Single-domain antibodies (nanobodies) with neutralizing ability have also garnered interest with regard to the development of anti-SARS-CoV-2 therapies. Nanobodies derived from the heavy chain-only subset of camelid immunoglobulins have multiple advantages over conventional Abs. These include their smaller size and higher stability, which promise compatibility with inhalatory administration and high local drug concentration and bioavailability, and a simpler structure, which enables manufacturing by readily available microbial systems [43]. Nanobodies (a.k.a., single-domain antibodies (VHHs)) can be isolated from immunized llamas [44,45], alpacas [46–48] or mice engineered to produce VHHs cloned from alpacas [47], then selected through a yeast surface-display library [49], synthetic nanobody libraries [50] or phage display technologies [51]. Cocktails consisting of nanobodies recognizing non-overlapping epitopes are expected to show improved potency [48]. Rapid and low-cost development may make it possible to quickly produce SARS-CoV-2 variant-specific nanobodies to combat escape mutations [51].

### Discovery of small-molecule drugs for SARS-CoV-2

**Figure 4.**
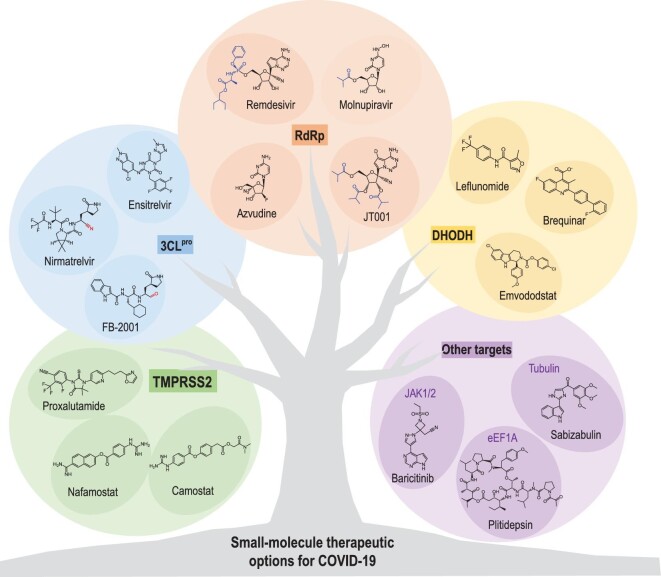
Chemical structures of small-molecule drugs and candidates in clinical trials, and their targets for the treatment of COVID-19.

Six human coronaviruses have been identified so far, including the agent causing the common cold, but no anti-coronaviral treatment had entered clinical trials before the emergence of SARS-CoV-2 [52]. The outbreak of COVID-19 makes it clear that effective therapeutics are crucial when preventive measures are missing. Tremendous effort has poured into anti-SARS-CoV-2 drug discovery, especially with regard to developing small-molecule drugs, which have substantial advantages, in terms of cost, production, stability, distribution and administration, compared with biologics. High-throughput technologies have played a key role in assisting the screening of small-molecule antiviral drugs, the characterization of drug antiviral activities, and the demonstration of the mechanism of action (MOA) of antiviral drugs. For example, proteomics and transcriptomics play a key role in identifying the dysregulated pathways, essential genes and proteins underlying the pathophysiology of COVID-19, which provides potential targets for drug development. Remarkably, a small number of direct-acting antivirals, including remdesivir [53], molnupravir [54] and nirmatrelvir [55], have received emergency approval (Fig. 4 and Table 3). Due to length constraints, here we will focus on small-molecule antivirals that target essential proteins in the viral life cycle, have shown significant *in vitro* and *in vivo* efficacy, and have been evaluated in clinical trials (Table 3).

**Table 3.** Small molecules authorized or in clinical trials for the treatment of COVID-19.

Name	Mechanism of action	Status	IC_50_ (μM)	EC_50_ (μM)	Indications/notes	Ref.
Remdesivir (Veklury^TM^; GS-5734; RDV)	RdRp inhibitor	Approved in US, the European Union, Japan, other (2020)	ND^a^	0.77 (Vero E6 cells)	Approved for the treatment of COVID-19 in adults and pediatric patients (12 years of age and older and weighing at least 40 kg) who (i) are hospitalized, or (ii) are not hospitalized and have mild-to-moderate COVID-19 and are at high risk of progression to severe COVID-19, including hospitalization or death.	[56,61,66,68]
Molnupiravir (Lagevrio^TM^; EIDD-2801; MK-4482)		Emergency use authorization (EUA) in UK, US, Japan, other (2021)	ND^a^	0.30 (Vero E6 cells)	UK: Indicated for the treatment of mild-to-moderate coronavirus disease 2019 (COVID-19) in adults with a positive SARS-CoV-2 diagnostic test who have at least one risk factor for developing severe illness. USA: Authorized for emergency use for the treatment of mild-to-moderate coronavirus disease 2019 (COVID-19) in adults with positive results of direct SARS-CoV-2 viral testing who are at high risk of progressing to severe COVID-19, including hospitalization or death, and for whom alternative COVID-19 treatment options approved or authorized by the US FDA are not accessible or clinically appropriate.	[54,69–71]
Azvudine		Approved in China (2022)	ND^a^	4.31 (Vero E6 cells) 1.2 (Calu-3 cells)	Approved number by CFDA: H20210036; H20210035.	[77]
JT001 (VV116; MINDEWEI^TM^)		EUA in Uzbekistan (2021), China (2023)	ND^a^	0.35 (Vero E6 cells)	Approved for the treatment of moderate-to-severe COVID-19 patients.	[179]
Nirmatrelvir/ Ritonavir (Paxlovid^TM^; PF-07321332/Ritonavir)	3CL^pro^ (M^pro^) inhibitor	EUA in US, UK, (2021), the European Union (2022)	0.00311^b^	0.0745 (Vero E6 cells)	Authorized for emergency use for the treatment of mild-to-moderate COVID-19 in adults and pediatric patients (12 years of age and older weighing at least 40 kg) with positive results of direct SARS-CoV-2 viral testing and who are at high risk of progression to severe COVID-19, including hospitalization or death.	[55,82]
Ensitrelvir (Xocova^TM^; S-217622)		Approved in Japan under ERAS (2022)	0.013	0.37 (Vero E6 cells)	Approved for the treatment of mild-to-moderate COVID-19 patients, regardless of risk factors or vaccination status, during the Omicron-dominant phase of the epidemic. (https://www.shionogi.com/global/en/news/2022/11/e20221122.html)	[84]
SSD8432 (Simnotrelvir; XIANNUOXIN^TM^)		Approved in China (2023)	ND^a^	ND^a^	Approved for the treatment of adult patients infected with mild-to-moderate COVID-19. Chemical structure and reference related to COVID-19 of SSD8432 are not available.	
Lufotrelvir (PF-07304814)		Phase III (NCT04501978)	0.00027^b^	ND^a^	Status: Discontinued (Pfizer). **NCT04501978**: ACTIV-3: Therapeutics for inpatients with COVID-19 (TICO) (University of Minnesota).	[180,181]
FB-2001 (Bofutrelvir; DC-402234; 11a)		PhaseⅡ/III (NCT05445934)	0.053	0.53 (Vero E6 cells)		[63]
PBI-0451		Phase II (NCT05543707)	ND^a^	ND^a^	Chemical structure and reference related to COVID-19 of PBI-0451 are not available.	
EDP-235		Phase I (NCT05246878)	ND^a^	ND^a^	Chemical structure and reference related to COVID-19 of EDP-235 are not available.	
Baricitinib (Olumiant)	JAK inhibitor	EUA in US (2021)	0.0059 (JAK1) 0.0057 (JAK2)	ND^a^	For emergency use by healthcare providers for the treatment of COVID-19 in hospitalized adults and pediatric patients 2 years of age or older requiring supplemental oxygen, non-invasive or invasive mechanical ventilation, or extracorporeal membrane oxygenation (ECMO).	[86,88]
Sabizabulin (ABI-231; APP-111; VERU-111)	α- and β-tubulin	Phase III (NCT04842747)	ND^a^	ND^a^	Reference related to COVID-19 of Sabizabulin is not available. A request for FDA EUA was submitted on 7 June 2022 (https://verupharma.com/pipeline/sabizabulin-for-covid-19/).	[89]
Plitidepsin	eEF1A inhibitor	Phase III (NCT04784559)	ND^a^	0.0007		[90]
Camostat mesylate (DWJ1248)	TMPRSS2 inhibitor	Phase III (NCT04713176; NCT04721535)	0.0062	0.087 (Calu-3 cells)	**NCT04713176**: Efficacy and safety of DWJ1248 with Remdesivir in severe COVID-19 patients. **NCT04721535**: A study of DWJ1248 in prevention of COVID-19 infection after exposure to SARS-CoV-2.	[92–94]
Nafamostat mesylate (CKD-314)		Phase III (NCT04390594; NCT04483960; NCT04871646)	0.00027	0.005 (Calu-3 cells)	**NCT04390594**: Efficacy and safety evaluation of treatment regimens in adult COVID-19 patients in Senegal. **NCT04483960**: Australasian COVID-19 Trial (ASCOT) ADAptive Platform Trial (ASCOT ADAPT). **NCT04871646**: Phase III clinical trial to evaluate the efficacy and safety of CKD-314.	[93,94]
Proxalutamide (GT-0918)		EUA in Paraguay (2021)	ND^a^	ND^a^	Authorized for emergency use for the treatment of hospitalized patients with COVID-19 infections.	[182]
Leflunomide	DHODH inhibitor	Phase III (NCT05007678)	ND^a^	41.49 (Vero E6 cells)	/	[99,100,102]
Brequinar sodium		Phase II (NCT05166876)	ND^a^	0.123 (Vero E6 cells)	/	[100]
Emvododstat (PTC-299)		Phase II/III (NCT04439071)	ND^a^	0.0026 (Vero cells)	/	[85]
RP-7214		Phase II (NCT05007236)	ND^a^	ND^a^	Chemical structure and reference related to COVID-19 of RP-7214 are not available.	

^a^ND, Not determined. ^b^*K*_i_ values. ‘/’ indicates the drug has not been authorized in clinical trials.

#### Identification of RdRp inhibitors

The genomic RNA replication of CoVs in infected cells is mediated by replication-transcription machinery, of which RNA-dependent RNA polymerase (RdRp) is the core component [56,57]. RdRp catalyzes the synthesis of a nascent RNA strand complementary to a given RNA template in many viruses, including CoVs, the hepatitis C virus (HCV), influenza and Zika. RdRp displays a highly conserved catalytic domain, making it an excellent target for antiviral drugs, especially nucleotide analogue prodrugs. Molecules such as favipiravir, ribavirin, sofosbuvir, baloxavir and dasabuvir, are nucleotide analogue prodrugs, which are marketed as antiviral drugs for several viral infections [58]. Given the significance of the target and the widespread clinical application of nucleotide analogue antivirals, drug repurposing has emerged as a viable method for the quick discovery of possible COVID-19 therapies (Fig. 4 and Table 3). SARS-CoV-2 replication has reportedly been halted by a number of approved drugs [59,60]. Several of these drugs have gone through clinical trials to assess whether they may be effective against COVID-19 and the SARS-CoV-2 infection.

Remdesivir (GS-5734) was one of the first antiviral drugs to demonstrate *in vitro* efficacy against SARS-CoV-2 (EC_50_ : 0.77 μM) [61], attracting considerable attention right from the start of the COVID-19 pandemic. Gilead Sciences initially developed the drug for HCV infections and then expanded its use to Ebola and Marburg virus infections [62]. Remdesivir is a phosphoramidate prodrug of an adenine derivative (GS-441524), which is converted intracellularly into a triphosphate form (GS-443902). GS-443902 competes with adenosine nucleoside triphosphates for incorporation into the viral RNA by RdRp, causing premature termination of the viral RNA chain [63]. To profile the metabolic pathway of remdesivir, the mRNA expression of nucleoside metabolic enzymes was analyzed using public human-lung single-cell bulk mRNA sequence data sets [64]. The results indicated that carboxylesterase 1 (CES1), cathepsin A (CatA) and histidine triad nucleotide-binding protein 1 (HINT1) were involved in the transformation of remdesivir into a monophosphate form, providing a better understanding of the MOA of remdesivir and assisting the discovery of novel nucleotide analogs as antiviral drugs. Due to the high conservation of CoV RdRp non-structural protein (nsp)12 between different coronaviruses, especially within genogroups, remdesivir has shown broad-spectrum antiviral activity against several viruses, including Ebola virus, SARS-CoV and MERS-CoV, in both cultured cells and animal models [65–67]. While preliminary evidence suggested that remdesivir could slow the progression of COVID-19, larger randomized trials failed to demonstrate clear benefits in reducing mortality in hospitalized patients [68]. Despite the inconsistent clinical outcomes, remdesivir has received EUA in several countries to treat COVID-19 and has been approved by the US FDA for use in hospitalized patients with severe COVID-19 [69].

In 2021, molnupiravir (MK-4482; EIDD-2801), an isopropyl ester prodrug of β-D-N^4^-hydroxycytidine (NHC), also received EUA from the US FDA as an oral drug [70]. One of the key benefits of oral medications is that they allow for the early prescription of antiviral drugs without the need for hospitalization. Indeed, molnupiravir elicited significant improvement in hospitalization risk and mortality in non-hospitalized COVID-19 patients [71]. The drug was specifically developed against influenza [72], but revealed broad-spectrum antiviral activity against CoVs [54]. Next-generation sequencing (NGS) analysis of the 538-base-pair (bp) region of the viral genome in the nsp15 endonuclease revealed a significantly increased error rate in NHC-treated MERS-CoV RNA, while NHC antiviral activity was associated with increased viral mutation rates. Molnupiravir treatment and prophylaxis reduced the replication and pathogenesis of SARS-CoV-2 in the human lung-only (LoM) humanized mouse model (the similarity between the LoM model and human SARS-CoV-2 infection has been demonstrated by RNA-seq analysis) [73] and also blocked transmission of the virus to untreated ferrets [74]. Unlike remdesivir, the MOA of molnupiravir is to promote mutations in the replication of viral RNA. Tautomers of NHC can mimic both cytidine (C) and uridine (U), thereby allowing the triphosphate form of NHC to be effectively incorporated into nascent viral RNA by RdRp. This results in increased mutagenesis when the NHC-containing RNA strand is used as a template to form NHC-A or NHC-G base pairs [75]. The cumulative mutations from repeated replication cycles can lethally alter the genetic composition of the virus. However, the possibility of increased viral mutagenicity raises concerns about the potential consequent risk of the development of viral mutants resistant to therapeutics.

Azvudine (FNC) is an HIV-1 RdRp inhibitor that received approval from the NMPA for AIDS treatment in 2021 [76]. The drug demonstrated micromolar potency against SARS-CoV-2 in cell lines, while oral administration of azvudine in SARS-CoV-2-infected rhesus macaques showed promising results, as evidenced by significant anti-SARS-CoV-2 activity in the thymus [77]. Analysis of single cells from thymic samples showed improvements in immune cells in the thymus in the FCN-treated group, including an increase in viable CD4^+^, CD8^+^ T cells, B and NKT cells. Further, gene enrichment analysis of the differentially expressed genes (DEGs) in thymic immune cells revealed that FNC significantly enhanced pathways involved in immunity, anti-viral response, inflammation, innate immunity and T cell activation, with CD4^+^ cells being mainly involved. The thymus may be a key organ for controlling COVID-19, so the chemo-to-immune antiviral MOA of FNC may be a reasonable strategy in the development of anti-SARS-CoV-2 drugs. Azvudine has undergone a randomized, single-arm clinical trial for compassionate use, in which patients quickly and completely achieved negative conversion of SARS-CoV-2 nucleic acid when it was taken orally [77]. The drug has been approved by the NMPA for the management of COVID-19 in China.

VV116 (JT001) is a tri-isobutyrate ester of a 7-deuterated derivative of GS-441524 that was developed after the outbreak of COVID-19 [78]. The hydrobromide salt form of VV116 exhibited a remarkably improved oral bioavailability (F = ∼80% in rats) and a favorable tissue distribution profile, allowing oral administration of VV116 in both preclinical and clinical studies [79]. In a panel of SARS-CoV-2 variants (Alpha, Beta, Delta and Omicron), VV116 had strong cellular activity, and was therapeutically effective in SARS-CoV-2-infected mice [78]. In December 2021, Uzbekistan granted EUA approval for VV116, which has also been approved by the NMPA in China for the treatment of COVID-19.

#### Identification of 3CL^pro^ inhibitors

Upon entry into the host cell cytoplasm, the viral RNA is uncoated and translated into two polyproteins, pp1a and pp1ab [80], which are essential for the production of new mature virions. These proteins are extensively processed by the predominant viral protease (M^pro^, also called chymotrypsin-like cysteine protease, 3CL^pro^) along with papain-like protease (PL^pro^) to generate 16 nsps [52]. Researchers have delineated the human protein substrate landscape of 3CL^pro^ using proteomics and substrate screening techniques. There were at least 101 human substrates for 3CL^pro^, some of which were involved in RNA processing, translation and cell cycle control [81].

**Figure 5.**
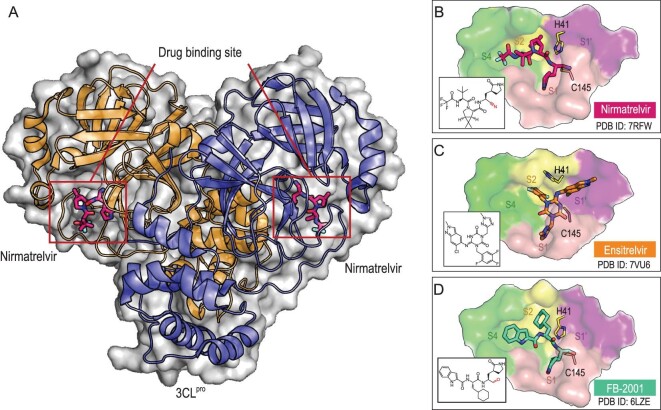
Crystal structures of SARS-CoV-2 3CL^pro^ in complex with inhibitors. (A) The dimeric 3CL^pro^ with bound nirmatrelvir. The binding modes of nirmatrelvir (B), ensitrelvir (C) and FB-2001 (D) in the active site (S1–S4 subsites) of 3CL^pro^. Adapted with permission from [80].

3CL^pro^, which primarily exists as a catalytically active dimer, is unique in recognizing the rigorous substrate specificity and cleavage at the P1-Gln position. Since human proteases lack this characteristic, it might be possible to develop selective protease inhibitors with favorable clinical safety. Unlike RdRp inhibitors, 3CL^pro^ inhibitors, such as nirmatrelvir, ensitrelvir and FB2001, have advanced into clinical trials. These inhibitors have been *de novo* developed using a structure-based design strategy instead of drug repurposing (Figs 4 and 5, and Table 3). Nirmatrelvir (PF-07321332) is the first novel inhibitor of SARS-CoV-2 3CL^pro^ granted an EUA by the US FDA (in December 2021) for treating mild-to-moderate COVID-19 patients with a minimum age of 12 [82] (Fig. 4 and Table 3). Nirmatrelvir is a peptidomimetic inhibitor of SARS-CoV-2 3CL^pro^ that has a glutamine-like pyrrolidone in the P1 position, which fits well into the highly conserved S1 subsite, and a nitrile as an electrophilic warhead to form a reversible covalent connection with the catalytic cysteine [55] (Fig. 5A and B). Additionally, a fused cyclopropyl ring with two methyl groups, a *tert*-leucine and trifluoroacetamide engage the S2, S3 and S4 subsites of the protease, respectively, increasing the binding affinity of nirmatrelvir to the target. Nirmatrelvir demonstrated an excellent selectivity profile, and potently inhibited SARS-CoV-2 3CL^pro^ with an IC_50_ of 7.5 nM and an EC_50_ of 74.5 nM in Vero E6 cells treated with an efflux inhibitor. Nirmatrelvir is sold as Paxlovid (oral pills) and is administered in combination with a low dose of ritonavir to maintain a high level of the drug in the body. Ritonavir is a potent inactivator of CYP3A4, a cytochrome enzyme critical to drug metabolism [55]. In a phase II–III clinical trial, Paxlovid significantly reduced hospitalization or death by 88% compared to a placebo among patients who did not receive COVID-19 therapeutic mAb treatment, representing a promising therapeutic treatment for COVID-19 [83].

Ensitrelvir (S-217622), the first non-peptidomimetic, non-covalent inhibitor of SARS-CoV-2 3CL^pro^ (Fig. 5C), was *de novo* developed via high-throughput mass-spectrometry-based virtual screening followed by structure-guided optimization [84]. The drug exerted potent inhibition of SARS-CoV-2 3CL^pro^ with an IC_50_ of 13 nM, antiviral activity (EC_50_ = 0.37 μM) and favorable pharmacokinetics profiles. The excellent pharmacokinetic properties of ensitrelvir allow it to be orally administered without ritonavir so as to avoid the drug–drug interactions caused by ritonavir. Moreover, ensitrelvir exhibited potent cellular activity against seven VOCs, including the Alpha, Beta, Gamma, Delta, Omicron, Lambda and Mu strains. Oral administration of ensitrelvir dose-dependently reduced intrapulmonary viral titer in SARS-CoV-2-infected mice and hamsters. These favorable preclinical data prompted the progression of ensitrelvir into a phase III clinical trial for non-hospitalized patients with COVID-19. Ensitrelvir (Xocova^TM^) was approved in Japan for the treatment of COVID-19 under the emergency regulatory approval system (ERAS) in November 2022.

FB2001 is also a covalent peptidomimetic inhibitor of SARS-CoV-2 3CL^pro^ with an aldehyde as the warhead (Fig. 5D) [63]. The drug potently inhibited the protease, with an IC_50_ of 53 nM, as well as the replication of SARS-CoV-2 in Vero E6 cells, with an EC_50_ of 0.53 μM. Moreover, FB2001 exhibited favorable pharmacokinetic properties and low toxicity when administered intravenously. Currently, a phase Ⅱ/III clinical study is underway to evaluate the efficacy of FB2001 in non-hospitalized patients with COVID-19 infection. Besides nirmatrelvir, ensitrelvir and FB2001, three SARS-CoV-2 3CL^pro^ inhibitors, EDP-235, PBI-0451 and SSD8432 (SIM0417), whose chemical structures and antiviral activity data are not disclosed, are also under phase I, II and II/III clinical trials, respectively. In particular, SSD8432 (simnotrelvir) in combination with ritonavir (XIANNUOXIN^TM^) was conditionally approved by the NMPA on 29 January 2023.

#### Host targeting antiviral agents

Aside from virus-targeting drugs, host-targeting antiviral agents offer promising COVID-19 therapeutic options by directly preventing viral replication or regulating the host inflammatory response (Table 3). By applying tissue-specific Mendelian randomization (MR) of gene expression and plasma protein levels, the causal effects of drug targets on 49 viral infection phenotypes, 501 complex diseases and 72 disease-related phenotypes were estimated, and a prioritization approach was used to further screen drugs with potential for COVID-19 treatment [85]. Baricitinib, a JAK inhibitor previously approved to treat rheumatoid arthritis [86], effectively suppressed elevated cytokine levels in severe SARS-CoV-2 infection and was predicted to inhibit the cellular entry of SARS-CoV-2 [55,87]. In November 2020, the US FDA issued an EUA for the treatment of suspected or laboratory-confirmed COVID-19 in hospitalized adult and pediatric patients needing supplemental oxygen [58], due to the drug producing a considerable reduction in mortality [88]. Sabizabulin, an oral small-molecule inhibitor of tubulin polymerization [89], can interfere with the transport of androgen receptors (ARs) into the cell nucleus as well as the transport of the virus within the cell by microtubules. Sabizabulin can also inhibit the release of inflammatory cytokines. It is currently in phase III clinical evaluation as a therapeutic treatment for hospitalized moderate-to-severe COVID-19 patients at high risk of acute respiratory distress syndrome. Through inhibition of eukaryotic translation elongation factor 1A, plitidepsin (aplidin) demonstrated extremely potent activity against SARS-CoV-2—27.5-fold greater potency than that of remdesivir [90]. Plitidepsin is in phase III clinical evaluation as a therapeutic treatment for hospitalized moderate-to-severe COVID-19 patients at high risk of acute respiratory distress syndrome.

Proteomics data revealed that serine protease transmembrane protease serine 2 (TMPRSS2) is required to activate the fusion of viral and host membranes through cleavage at the S1–S2 junction of the S glycoprotein, thus identifying this serine protease as an attractive therapeutic target [91]. Two approved serine protease inhibitors, camostat and nafamostat, were repurposed as anti-SARS-CoV-2 agents [92,93] (Fig. 4 and Table 3). Camostat is used to treat chronic pancreatitis and postoperative reflux esophagitis in Japan. Early in the COVID-19 pandemic, camostat attracted particular interest owing to its potent activity against the TMPRSS2-dependent entry of SARS-CoV-2 [92]. Nafamostat is an approved anticoagulant in Japan and Korea. The inhibitory activity of nafamostat against TMPRSS2 was shown to be ∼10 times greater than that of camostat [94]. More importantly, nafamostat treatment resulted in a significant reduction of viral titers of SARS-CoV-2 in mice [95].

Notably, proxalutamide, an antagonist of AR, underwent clinical trials for its effectiveness in treating COVID-19 and received an EUA in Paraguay for treating hospitalized COVID-19 patients [96,97] (Table 3). A possible mechanism of proxalutamide's antiviral action is associated with its effect on TMPRSS2 protein expression. Viral replication promotes *de novo* pyrimidine biosynthesis in the host. Consequently, human dihydroorotate dehydrogenase (DHODH), a key enzyme catalyzing a rate-limiting step in pyrimidine biosynthesis, has become a therapeutic target of interest [98,99]. Leflunomide, a DHODH inhibitor used clinically to treat autoimmune diseases, demonstrated broad-spectrum antiviral activity. Other DHODH inhibitors, such as brequinar [100] and emvododstat [85], inhibit SARS-CoV-2 replication in Vero E6 cells in the nM to μM range. Of note, DHODH inhibitors can synergize with nucleoside analogs, such as remdesivir and molnupiravir, to block SARS-CoV-2 replication [101]. A small-scale clinical study enrolling 10 hospitalized COVID-19 patients revealed that leflunomide was superior to placebo treatment in shortening viral shedding time [102]. These inhibitors, including RP-7214, are currently in phase II or III trials for treating COVID-19.

**Figure 6.**
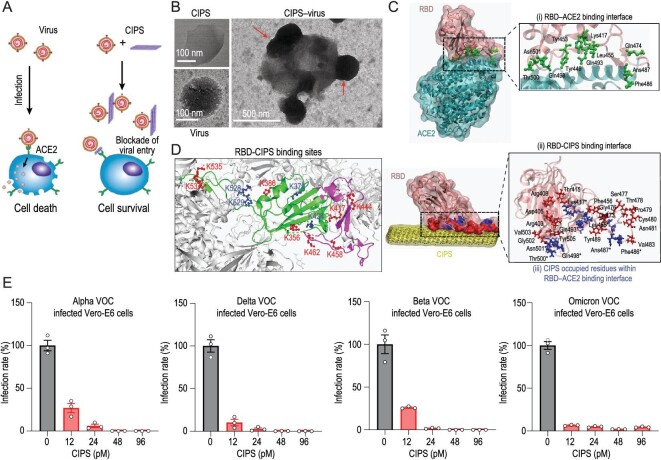
Antiviral effect of CIPS against SARS-CoV-2. (A) A schematic depiction of CIPS’s ability to prevent SARS-CoV-2 infection of target cells. (B) Transmission electron microscope (TEM) images showing the interaction between SARS-CoV-2 and CIPS. The red arrows indicate virus particles adhering to CIPS. (C) MD simulation of the ACE2–RBD and CIPS–RBD binding interfaces. (D) Schematic diagram of the binding interface between CIPS and the RBD of the spike protein, S1. The S1 conformation is shown in grayscale, while the RBD is shown in color. The RBD encompasses two regions in the RBD–CIPS interaction: the N437-Y508 region (purple), which shows the strongest binding efficiency, and the remaining region (green). (E) CIPS inhibits the *in vitro* infectivity of SARS-CoV-2 VOCs. Adapted with permission from [106].

Nanotechnology has also played a pivotal role in the fight against SARS-CoV-2 in various capacities. Through their interactions with viral surface proteins, nanomaterials can prevent effective virus–host cell contact. An inhalable nanocatcher, soluble human ACE2 produced by genetically engineered cells [103], was developed to inhibit host cell infection by competing with SARS-CoV-2 binding. By introducing a mucosal adhesion excipient, hyaluronic acid (HA), the nanocatcher was able to significantly prolong retention time in the lung and enhance viral inhibition in a mouse model. In addition, the presence of sucrose significantly improved the nanocatcher's storage and transport stability [104]. Another group constructed a nanodecoy by fusing cell membrane nanovesicles derived from highly expressed hACE2 cells and human monocytes, which display abundant cytokine receptors. The nanodecoy was able to neutralize the virus and inflammatory cytokines at the same time in a mouse model, effectively disrupting SARS-CoV-2 infection [105]. Zhang *et al.* described ultrathin, two-dimensional CuInP_2_S_6_ (CIPS) nanosheets as a new agent, able to inhibit SARS-CoV-2 infection *in vitro* and *in vivo* [106] (Fig. 6A and B). Mass spectrometry (MS)-based two-step isotope labeling lysine reactivity profiling (TILLRP) and molecular dynamics (MD) simulation were used to identify the binding sites between the virus RBD and CIPS, revealing that binding to CIPS engages the same RBD residues involved in ACE2 binding (Fig. 6C and D). Notably, CIPS can selectively and efficiently bind to the S glycoprotein of both wild-type SARS-CoV-2 and all VOCs (Fig. 6E). Most importantly, adhesion to CIPS not only blocks interaction with ACE2 and subsequent infection, but it also substantially increases the size and shape of the virus, which can then be readily taken up by macrophages and degraded within phagolysosomes. Overall, the study provides new ideas and strategies for the development of broad-spectrum antiviral drugs.

In general, the use of omics technologies has aided in the screening of drug targets, in the analysis of the biological relationships between targets and diseases, in evaluating the efficacy and MOAs of small-molecule drugs, and in exploring the broad spectrum of antiviral drugs. Biobanks built from omics strategies may become critical resources for rapidly screening small-molecule antiviral drugs in epidemic and pandemic situations in the near future.

### Design of COVID-19 vaccines

**Figure 7.**
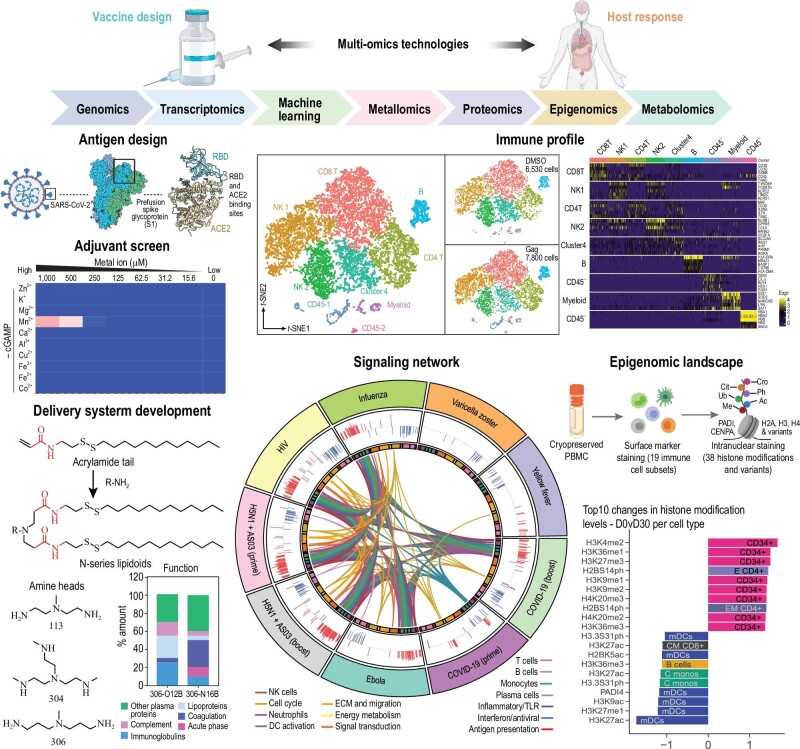
Omics science and the flow of biological information can assist in vaccine development. Created by Biorender.com. Reproduced with permission from refs [107–111].

To date, there are 50 authorized vaccines for COVID-19 and 242 in clinical or preclinical development. Several vaccines, including ChAdOx1 nCoV-19 (AstraZeneca/Oxford), Ad26.COV2.S (Janssen), NVX-CoV2373 (Novavax), BNT162b2 (Pfizer-BioNTech) and mRNA-1273 (Moderna), administered by intramuscular injection, have been granted EUA in many countries, with hundreds of millions of doses given worldwide. Despite the unsurpassed progress we have made in vaccine development, we still struggle to obtain a comprehensive understanding of the profiles of anti-SARS-CoV-2 immunity triggered by the different vaccines, and we still need to satisfactorily address the issue of vaccination against the emerging viral VOCs. Many multi-omics approaches, including glycomics, transcriptomics and proteomics, provide potential value in finding the characteristics of pathogens and elucidating host responses to vaccines, which can ultimately lead to a roadmap for vaccine design (Fig. 7, Table 4).

**Table 4.** The application of omics in vaccine development.

Application	Formulation and administration	Methodology	Details	Ref.
	RBD	Proteomics	pSer^a^-modified RBD stably combined with alum to obtain enhanced immunogenicity and humoral immunity.	[183]
	RBD	Proteomics	Self-assembling trimeric (RBD-I53-50A) and pentameric (I53-50B) RBD antigens into nanoparticles, eliciting more potent neutralizing antibody responses.	[184]
**Antigen design**	S glycoprotein	Immunoinformatics	Predicting T- and B-cell epitopes of the SARS-CoV-2 S glycoprotein.	[185]
	_	Immunoinformatics	Predicting potential immunodominant epitopes from the SARS-CoV-2 proteome.	[113]
**Adjuvant screening**	AS03	Luminex assay and systems serology	RBD-NP-AS03 stimulated a mixed T_H_1–T_H_2 response.	[119]
		Epigenomics and transcriptomics	AS03-adjuvanted influenza vaccine induces durable epigenomic changes in myeloid cells, leading to protection against viruses.	[111]
			AS03-adjuvanted RBD-I53-50 nanoparticle vaccine elicited durable and cross-protective immunity.	[120]
	CpG1018-alum	Luminex assay and systems serology	RBD-NP-CpG1018-alum stimulated a T_H_1-biased response.	[119]
	Alum	Luminex assay and systems serology	RBD-NP-alum stimulated a T_H_2-biased response.	[119]
	Alum pickering emulsion		Alum-stabilized pickering emulsion induced a strong increase of humoral and cellular immune responses.	[123]
	MPL	Luminex assay	For mRNA vaccine, TLR 7/8 ligand adjuvant stimulated high antiviral immune response.	[110]
	R848			
	3M-052		3M-052 promoted magnitude and durability of antibody responses via robust stimulation of innate immunity and bone marrow resident long-lived plasma cells.	[134,186]
	AS01		AS01 stimulates a T_H_1-biased immune response through caspase 1 activation.	[130]
	AS04		AS04 promotes TLR4 activation on innate cells.	[127]
	STING agonist	Proteomics	Using thermal shift assay to investigate the effect of metal ions on the activation of the STING pathway.	
		Transcriptomics	Using genome-wide transcriptomic analysis to identify 2′3′-cGAMP-regulated genes.	[107]
		Transcriptomics	Using RNA-seq^b^ analysis to show that STING agonist (diABZI) leads to activation of genes associated with the anti-COVID-19 IFN pathway.	
		Transcriptomics	Splenocyte transcriptomes indicate that the MnARK nanovaccine enhances immune responses via the cGAS-STING pathway.	[137]
**Delivery system development**	LNP	Proteomics	Detecting the protein corona by proteomics to design lung-targeting LNP.	[108]
		Luminex assay	LNPs amplified IL-1 activation without activating the IL-1Ra feedback.	[146]
**Vaccination route**	Intramuscular	Proteomics	Using Fc array to analyze the different humoral immune-specific antibody features induced by different vaccination routes. Using machine-learning approaches to predict the mechanism of antibody-related prevention of viral infection of different vaccination routes.	[149]
	Inhalation		Adv-nCoV inhalation vaccine induced higher IgG and IgA levels and T_H_1-type cellular immune response.	[150]

^a^pSer: phosphoserine; ^b^RNA-seq: RNA sequencing.

#### Antigen discovery and development

Omicron variants have now emerged as the predominant circulating strains on most continents, as determined by prompt application of NGS techniques, which have also provided crucial information on specific mutations [112,113]. Considering that multiple mutations in the SARS-CoV-2 variant sequence lead to changes in pathogenicity, infectivity, transmissibility and/or antigenicity [21], vaccines’ efficacy against variants has become an urgent problem [114]. Glycomics analysis of SARS-CoV-2 has received considerable attention during the pandemic and has contributed to the development of vaccines. Watanabe *et al.* revealed the glycosyl composition of 22 glycosylation sequons by site-specific MS (i.e. showing that the glycosyl groups at N234 and N709 were mainly of the oligomannose type) across the trimeric S structure, providing a benchmark for assessing the quality of immunogens in vaccines [115]. Using ultra-high-performance liquid chromatography (UHPLC), Brun *et al.* compared the glycan signatures of virus-derived stabilized recombinant trimer and non-stabilized S protein. The results showed that a stabilized trimeric prefusion S protein, accompanied by abolishing the furin cleavage site, may be likely to elicit desirable Ab responses [116]. Apart from the N-linked glycosylation landscape, 17 O-glycosylation modification sites in the S protein were identified for the first time using MS-based glycosylation identification technology. Researchers have found that 11 out of these 17 sites are located near glycosylated asparagine (Asn). Furthermore, an ‘O-Follow-N’ rule has been proposed whereby the O-glycosylation occurs near the site of N-glycosylation. This rule may be applicable to other proteins and promote immunogen design [117].

For assessing the features of protective immunity and vaccine efficacy, Pulendran's group devised and applied a ‘system vaccinology’ approach for the identification of protective epitopes tailored for different diseases and human populations [118]. Regarding SARS-CoV-2, the authors extensively applied omics tools to identify immunogenic epitopes of SARS-CoV-2 by defining epitopes recognized by B and T lymphocytes and epitopes associated with protective immunity in response to either infection or different vaccines, and validated the vaccines experimentally [119,120].

#### Adjuvant screen and design

As critical components of most vaccines, adjuvants are central to vaccine efficacy and durability, which is necessary for the establishment and amplification of protective adaptive immunity. In general, while adjuvants must induce inflammation to be effective, their inflammatory effects must be spatiotemporally controlled in order to avoid pathological tissue destruction. This must be considered when designing new adjuvants.

Aluminum salts, in their particulate form (alum), are the oldest and most commonly used and effective adjuvants. Aluminum-based adjuvants activate the nucleotide oligomerization domain (NOD)-like receptor thermal protein domain associated protein 3 (NLPR3) inflammasome and the production of IL-1β, thereby inducing a localized inflammatory reaction that amplifies the vaccine-induced specific adaptive immunity and long-term immunological memory [121]. Alum can function as an adjuvant through the depot effect, i.e. by increasing the time the antigen remains at the immunization site [122]. The approved inactivated SARS-CoV-2 vaccine, CoronaVac, formulated with an alum adjuvant, is well tolerated, safe and induces humoral responses (NCT04884685, NCT04352608). Since antiviral immune protection relies not only on the production of specific Abs but, more importantly, on the generation of specific cytotoxic T cells, it is particularly important to adjuvant design that the compound can also promote the generation of T-cell-based immunological memory. As alum preferentially amplifies humoral immune responses (production of Abs), an emulsion of alum admixed with squalene/water (a very effective clinically approved adjuvant) has been described [123]. PAPE (alum-stabilized pickering emulsion) exhibits a favorable biosafety profile and the capacity to induce strong humoral and cellular immune responses. A nitrogen bisphosphonate–modified zinc-aluminum hybrid adjuvant (FH002C) induced SARS-CoV-2 specific T cell responses in mice in combination with an S protein-based vaccine [124]. In FH002C, nitrogen bisphosphonate (risedronate), a drug for osteoporosis, was loaded onto alum by harnessing the alum's phosphophilic nature. FH002C also incorporates zinc ions, which have an immunostimulatory effect and promote T_H_1 responses [125]. A subunit vaccine adjuvanted with FH002C promoted the development of T_fh_ and germinal center B (GCB) cells and showed generation of antibody-producing plasma cells in the early days after immunization.

More recently, oil-in-water emulsion adjuvants, such as MF59, AS01, AS03 and AS04, and molecular adjuvants, such as CpG ODN (oligonucleotide), have been used in US-FDA-approved human vaccines. In contrast to alum, the adjuvant effect of MF59 seems to depend on the activation of the inflammatory signaling adapter protein, MyD88 (myeloid differentiation primary response 88) but is independent from NLRP3 inflammasome activation [126]. MF59 induces a transient release of adenosine triphosphate (ATP) from injected muscle, which is likely related to the stimulation of adaptive immune responses [121]. Recently, Kim *et al.* demonstrated that MF59 stimulated CD8 T cells, but not antibody responses, through a RIPK3-dependent pathway [126]. AS0 adjuvant systems have been developed through a rational combination of classical adjuvants, including alum, emulsions and liposomes, with immunostimulatory molecules, such as MPL and QS21. AS04 is composed of MPL (3-*O*-desacyl-4′-monophosphoryl lipid A) and aluminum salts. Through Toll-like receptor 4 (TLR 4) activation of innate cells, the protection induced by the AS04-based human papillomavirus (HPV)-16/18 vaccine remains 100% complete for as long as nine years after immunization [127]. Immunization with poly (D, L-lactic-co-glycolic acid) (PLGA) particles containing MPL and R848 (the TLR7 ligand, resiquimod) was able to induce a molecular profile (based on RNA-seq) characteristic of activated B cells and early programming of B cell memory, suggesting that the combination of TLR ligands can promote memory B cell generation [128]. AS03 is a squalene-in-water emulsion (as MF59) with the addition of α-tocopherol. AS03 is being evaluated in COVID-19 vaccine clinical trials. Given the similarity of MF59 and AS03, it is likely they engage common innate immune activation pathways [129]. AS01 is a unique combination of MPL and QS-21, a known adjuvant extracted from *Quillaja saponaria* [130], formulated within liposomes. AS01 exhibits an excellent adjuvant capacity for both humoral and cellular immune responses [130].

Considering the age-related decline of immune function, there is a need to develop adjuvants to increase immune responses in the elderly. CpG 1018, a TLR9 agonist, has been used as an adjuvant for the hepatitis B vaccine in older adults. This adjuvant has been found to be more effective than the alum-based hepatitis B virus (HBV) vaccine [131]. CpG 1018 is also currently being tested in clinical trials as an adjuvant for COVID-19 vaccines (NCT04982068 and NCT04990544). Besides overcoming the weaker immune responsiveness in the elderly [132,133], adjuvants may also help extend the duration of immune response from a vaccine. A novel TLR7/TLR8 adjuvant, 3M-052, elicited long-lasting protective immunity against HIV in a non-human primate model [134], inducing long-lived bone marrow plasma cells and increasing the expansion of T_FH_ cells, which persisted for 70 weeks. 3M-052, formulated with alum, is currently under evaluation as a vaccine adjuvant for HIV (NCT04177355).

In a search for new potent innate immune agonists, Li *et al.* screened 75 agonists by RNA-seq and identified stimulator of interferon gene (STING) agonists—cyclic dinucleotides (CDNs)—as agents able to induce high levels of interferons (IFNs), proinflammatory cytokines and chemokines in primary human respiratory epithelial cells and against SARS-CoV-2 *in vivo* in two different mouse models [135]. Based on a genome-wide transcriptomic analysis, Cai *et al.* identified cyclic dinucleotide 2′, 3′-cGAMP as a potent agonist of STING in *Drosophila*. Gene ontology (GO) enrichment analysis showed that 2′, 3′-cGAMP stimulates the Janus kinase-signal transducer and activator of transcription (JAK-STAT) pathway, further triggering a broad antiviral response [136]. To amplify STING activation, Sun *et al.* screened nutritional metal ions (Zn^2+^, Mn^2+^, Ca^2+^, K^+^, Co^2+^, Fe^3+^, etc.) and discovered that Co^2+^ and Mn^2+^ can augment the activity of STING agonists in type I IFN production [107]. As Mn^2+^ is already approved by the US FDA for use in pharmaceuticals, the authors assembled Mn^2+^ with CDN into nanoparticles, as potential adjuvants for enhancing immunity. Wang *et al.* used a new type of manganese nanoadjuvant in a SARS-CoV-2 subunit vaccine, which targets lymph nodes and elicits strong neutralizing responses against infection with pseudovirus and wild-type virus in a mouse model [137].

Stephenson *et al.* performed single-cell transcriptome analyses of peripheral blood mononuclear cells from 130 patients with COVID-19 and observed a significant decrease in IgA2 in symptomatic COVID-19, underscoring the importance of inducing an efficient mucosal immune response and the need for designing effective mucosal adjuvants [138]. A recent study showed that coadministration of alum with an elastase inhibitor elicited mucosal sIgA production [139]. A neutrophil elastase inhibitor promoted the expression of mucosal homing receptors and the trafficking of splenic B cells, indicating that inhibition of neutrophil elastase can improve the efficacy of alum-based vaccines. Wang *et al.* designed a mucosal adjuvant able to bypass the surfactant layer and reach alveolar macrophages. The authors assessed the adjuvant's ability to induce an anti-flu response in mice [140]. The mucosal adjuvant is a lung biomimetic nanoparticle (PS-GAMP) composed of cGAMP (2′,3′-cyclic guanosine monophosphate–adenosine monophosphate) and pulmonary surfactant (PS)-biomimetic nanoparticles. PS-GAMP was recognized and taken up by lung-resident alveolar macrophages and epithelial cells after intranasal administration, where STING was activated.

#### Vaccine delivery system development

**Figure 8.**
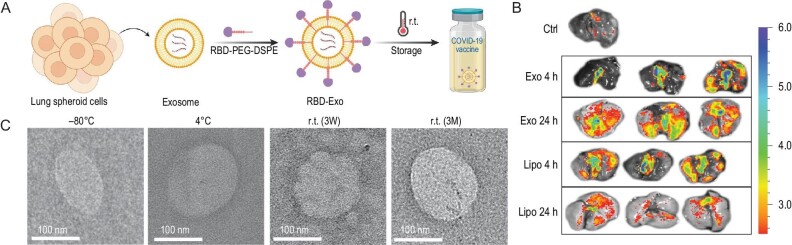
Exosomes decorated with a recombinant SARS-CoV-2 receptor-binding domain as an inhalable COVID-19 vaccine. (A) Schematic representation of the fabrication of the RBD-Exo vaccine. (B) *Ex vivo* imaging of mouse lungs 4 and 24 h after inhalation of fluorescent exosomes (Exo) or liposomes (Lipo). (C) TEM images of RBD-Exo after storage at −80°C, 4°C or r.t. for 3 weeks (3W) or 3 months (3M). Adapted with permission from [142].

Delivery systems play important roles in the development of an effective vaccine, especially for recombinant protein vaccines and messenger RNA (mRNA) vaccines. Delivery systems can co-deliver adjuvants and antigens, improve their stability, and increase the delivery to antigen-presenting cells, improving immune responses and the generation of immune memory [141]. Wang *et al.* used exosomes derived from human pulmonary globular cells as vaccine carriers, then conjugated the SARS-CoV-2 RBD protein onto the exosomal surface to prepare a virus-like particle vaccine (RBD-Exo) (Fig. 8) [142]. Inhalation of the RBD-Exo vaccine showed better lung retention and distribution than a liposome vaccine in a mouse model. Additionally, the vaccine stimulated mucosal immunity and T_H_1-biased T cell immunity in lung tissue, decreasing COVID-19-induced lung tissue damage. More importantly, the vaccine demonstrated excellent stability, allowing storage for three months at room temperature.

Messenger RNA (mRNA) vaccines represent one of the most significant innovations to emerge from the COVID-19 pandemic. According to the COVID-19 Vaccine Tracker (https://covid19.trackvaccines.org/), as of 24 November 2022, there were 64 mRNA vaccines under development worldwide and 9 approved, including two that have been included in the WHO Emergency Use Listing (EUL): BNT162b2, jointly developed by Pfizer/BioNTech, and mRNA-1273, developed by Moderna. The technical barriers to mRNA vaccines lie in sequence design, the control of mRNA stability, and delivery systems. The ideal delivery system must be safe, sufficiently stable and organ specific [143]. Lipid nanoparticles (LNPs) are the most clinically advanced mRNA carriers [144]. As of November 2022, almost all COVID-19 mRNA vaccines that are in development or that have received clinical approval use an LNP delivery system. LNPs have a variety of advantages for mRNA distribution, including standardized formation, flexibility, superior biocompatibility and substantial mRNA payload capacity. The challenge of optimal delivery to certain organs, in particular the respiratory mucosa, is crucial because the majority of LNPs target the liver.

Cheng *et al.* proposed a selective organ targeting strategy by varying the proportion of cationic and anionic lipids in LPN formulation [145]. In animal models, increasing the concentration of the anionic lipid, 18PA, encouraged targeting of the spleen, whereas increasing the concentration of the cationic lipid, DOTAP (1,2-dioleoyl-3-trimethylammonium-propane chloride), directed delivery to the lung. Qiu *et al.* achieved organ selectivity of LNPs *in vivo* by changing the connecting structure of the lipid tails [108]. Changing the linker from an ester bond (referred to as the O series) to an amide bond (referred to as the N series) shifted the specificity of mRNA delivery from the liver to the lung. Proteomic analysis revealed that the protein corona around LPNs changes significantly between 306-O12B and 306-N16 LNPs (14 of the 20 most abundant proteins differed between the formulations). Fibrinogen coating, which only occurred with 306-16B LNPs, is expected to improve endothelial cell adhesion, and is consistent with the observation of selective delivery of mRNA via 306-16B LNPs to lung endothelial cells. Lipid composition is a key element determining LNP tropism for different organs, and will be the basis for the rational design of selective organ/cell targeting of future LNPs.

A critical issue was recently brought to the public's attention: LNPs induce a strong inflammatory reaction in humans and not in mice, due to a species-specific difference in the control of IL-1-induced inflammation [146]. This finding stresses once more the need for relevant experimental models for vaccine development [147,148] and indicates the important need for the scientific community to accurately validate results and assess their relevance in real-life situations. In the case of LNPs, the ability to modify the lipid composition of the particles may aid in the development of carriers that induce the required controlled, local inflammation in human subjects without causing pathological effects.

#### Vaccination route selection

The induction of antigen-specific Abs and T cells and the establishment of long-term specific T and B cell memory are the main mechanisms forming the basis of protective vaccines. The nature and organ tropism of the targeted pathogen and the route of vaccine administration are important drivers of a successful vaccination. Abs, which are often taken as immunization benchmarks because they are easy to measure, use different mechanisms of attack on viruses and other infectious agents, from direct binding/neutralization to opsonization and facilitation of phagocytosis, complement-mediated lysis of infected cells, and Ab-mediated cellular cytotoxicity. Different classes of Abs with different properties and anatomical locations add to the complexity of the humoral response to infections/vaccines. Although the current trend is to deliver vaccines through the same route used by the natural infections (e.g. inhalation in the case of respiratory diseases) in order to achieve organ-selective ‘barrier’ immunization and memory, systemic immunization has been used successfully for a long time and has been shown to give rise to an immune profile different from that induced by the natural infection, but nevertheless efficient protection. An example is the study by Ackerman *et al.* in non-human primates vaccinated with an adenoviral vaccine against simian immunodeficiency virus (SIV) [149]. The authors found that Ab-dependent protection was achieved by different mechanisms with intramuscular delivery vs. inhalation (IgG-dependent phagocytosis by monocytes after intramuscular administration vs. IgA-dependent phagocytosis by neutrophils after vaccine inhalation) using functional and biophysical assessments and predictive hazard models. This confirms the notion that different immune mechanisms come into play depending on the location and mode of pathogen entry/encounter with immune effectors, but that the differing mechanisms are both able to attain protective immunity. However, the type of protective immunity can affect different outcomes. In the case of the current anti-COVID-19 vaccines, none are ‘sterilizing’ i.e. avoiding infection; vaccinated subjects can get infected, but their immunity is such that the infection is rapidly eliminated. Although vaccinated individuals do not develop the disease, or develop a mild form, these subjects can still transmit the infection to other subjects. Only vaccines that can stop the virus at its entry point (the respiratory system), e.g. inhaled mucosal vaccines that can specifically induce protective memory at the level of the respiratory mucosal tissue and the production of specific mucosal IgA Abs, can result in sterilizing immunity.

An adenovirus type 5 vector-based COVID-19 vaccine (Ad5-nCoV) developed by CanSinoBIO has been approved by the NMPA for emergency use as an inhalation booster immunization. The latest clinical trial results (NCT05043259) showed that the level of neutralizing Abs against SARS-CoV-2 were almost 20 times greater compared to an inactivated, homologous intramuscular booster [150]. In addition to greater IgA levels, the inhalation group exhibited a significantly induced T_H_1 type cellular immune response, which was unaffected by gender, age or other factors. Another study reported that intranasal enhancement with adenovirus vectors elicited higher levels of lung-resident memory T cells (T_RM_) compared with intramuscular injection of two doses of mRNA vaccine [151]. The transcriptional profile shows that the T_RM_ response lacks exhaustion characteristics, indicating that T_RM_ can offer long-term local protection against infection [152].

#### Dissection of mechanisms of vaccine efficacy

Multi-omics technologies are assisting us not only in understanding the host response to SARS-CoV-2 but also host responses to different vaccination regimens, thereby providing a solid foundation for the design of novel evidence-based vaccines with optimized antigens, adjuvants and administration routes, tailored to the different needs of different groups of people (such as children, the elderly, or patients with chronic diseases) [153,154].

A rigorous study on adaptive immune responses in subjects infected by SARS-CoV-2 showed the complex immune profiles of antiviral immunity and stressed the importance of T cells, in addition to Abs, in effective antiviral responses [155]. Activation of human dendritic cells (DCs) from 30 different donors was reported in response to 10 different adjuvants or candidate adjuvants, e.g. 7 Toll-like receptor (TLR) agonists, the classical aluminum hydroxide alum, the MF59 oil-in-water adjuvant Addavax^TM^, and Quil-A® [156]. Luminex xMAP technology and principal component analysis (PCA) were used for assessing the production of 25 cytokines and chemokines and grouping the adjuvants into strong, intermediate and weak categories. The strong adjuvants included the TLR agonists lipopolysaccharides (LPS) (TLR4 agonist, bacterial lipopolysaccharide, used as a positive control), MPL and R848. As expected, the viral-like agonist, R848, induced higher levels of IFNα and IL-1β, which are important for antiviral immune reactions, and a similar profile was obtained by lipid-complexed single-stranded RNA (which is also a virus-like TLR agonist). It is important to note that strong adjuvants, i.e. molecules that can induce a potent production of cytokines and chemokines, may have substantial side effects (e.g. cytokine storm). The most effective strong adjuvant, LPS, shows exceedingly potent inflammatory effects and toxicity in animals and human beings, and therefore is not clinically viable as an adjuvant.

The induction and regulation of epigenetic changes in innate immune cells has recently received renewed attention in the design of improved vaccines. Pathogens have long been known to induce epigenetic changes in innate immune cells, and the same has been observed with vaccines (such as Bacillus Calmette-Guérin (BCG)) and adjuvants [157]. A recent study utilized single-cell transposase-accessible chromatin with sequencing (scATAC-seq) to analyze the epigenomic profiles of immune cells in patients convalescing from COVID-19 [158]. The trained and activated monocytes, accelerated B cells, and expanded effector and memory CD8^+^ T cells were the main cells to establish immune memory via a remodeling of the chromatin accessibility landscape. That study facilitates our understanding of how memory cells are established and provides a new approach for adjuvant design. Another study mapped the single-cell epigenetic and transcriptomic profiles, in leukocytes of individuals vaccinated against influenza, using RNA-seq and scATAC-seq [111]. Epigenetic changes persisted for up to six months. Monocytes, the major innate immune effector cells, can be divided into different subpopulations according to the accessibility of AP-1 and the consequent upregulation of inflammation-related genes. The oil-in-water adjuvant, AS03, reduced histone acetylation, AP-1 accessibility and the expression of various TLR-related inflammatory factors, while increasing chromatin accessibility at the interferon regulatory factor (IRF) and signal transducer and activator of transcription (STAT) loci, leading monocytes to an antiviral state.

Modulation of epigenetic changes by vaccines and adjuvants is most likely fundamental to the establishment of innate memory, a mechanism of increased non-specific resistance to infections and other diseases triggered by previous exposure (e.g. vaccination) [159,160]. The example of BCG, a live vaccine against tuberculosis, is exemplary in that it shows that vaccination in children can non-specifically increase survival/resistance to diseases [161–163]. Not only can innate memory substantially increase vaccine efficacy by contributing to long-term resistance, but innate memory can also broaden vaccine coverage, achieving protection against unrelated or partially related infections. In mammals, innate memory based on epigenetic changes can last a lifetime and even be passed down to progeny [164], or it can be reprogrammed to adapt to newly incoming stimuli. In this context, epigenetic reprogramming-based adjuvants and vaccines can be developed to reprogram subjects with an ‘immunobiography’ that biases their immune reactivity toward insufficient protection to mount a protective response (e.g. elderly people) [165,166].

A recent study systematically analyzed the innate and adaptive immunity generated by mRNA vaccines [109]. Arunachalam *et al.* comprehensively analyzed the immune profiles of 56 healthy volunteers vaccinated with the BioNTech mRNA vaccine (BNT162b2), using a systems vaccinology approach. BNT162b2 vaccination produced large amounts of neutralizing Abs against wild-type SARS-CoV-2 and, to a lesser extent, against the B.1.351 VOC. Using whole blood for mRNA sequencing, gene set enrichment analysis (GSEA) showed that the booster vaccine produced a stronger immune response than the primary vaccination: (i) more specific multifunctional CD4^+^ and CD8 T^+^ cells were generated; (ii) higher concentrations of IFNγ were produced; (iii) dendritic cell activation was increased; and (iv) the proportion of inflammatory monocytes was up-regulated. Because mortality from COVID-19 is highest in the elderly, and the elderly population does not generally respond well to vaccination, the study examined whether there were age-related differences in response to mRNA vaccination. Changes in monocytes and inflammatory modules tended to be greater in younger participants, while expression of B and T cell modules was increased in older adults. The authors further examined transcriptional changes at the single-cell level by sequencing (CITE-seq (cellular indexing of transcriptomes and epitopes by sequencing)) 45 peripheral blood mononuclear cell samples from 6 individuals. Consistent with previous results, single-cell transcriptomic analysis showed that, after secondary immunization, mRNA vaccination uniquely induced a cluster of antiviral myeloid cells with an ∼100-fold increase in proportion; these cells were enriched in IFN-responsive transcription factors and had decreased AP-1. At the same time, the authors identified distinct immune pathways associated with CD8^+^ T cells and NAb responses, and showed that monocyte signatures were associated with NAb responses against the B.1.351 variant.

Another study compared the time-dependent development of the immune response in subjects vaccinated with different types of vaccines [120]. Although there was little overlap in day 1 responses to the first dose of BNT162b2 mRNA vaccine with other vaccines, day 1 post-booster responses were comparable to those of other adjuvanted vaccines (H5N1+AS03), live virus vaccines (Ebola and HIV vaccines) and inactivated vaccines. Common features include IFN signaling, dendritic cell activation and inflammatory responses. Cell cycle characteristics were linked to an upregulation of B cell and plasma cell modules for the majority of vaccinations, reflecting the growth of cells that secrete Abs.

## FUTURE PERSPECTIVES

Vaccines and antimicrobial drugs (Abs, antibiotics, antimicrobial peptides and small molecules) are the most effective ways to prevent and treat infectious diseases. For SARS-CoV-2 infection, several Ab therapies have been granted approval or emergency use by the US FDA. The use of novel small-molecule drugs, especially the oral formulations of molnupiravir and Paxlovid^TM^, have demonstrated significant therapeutic benefits in COVID-19 treatment, making an important step forward in the fight against SARS-CoV-2 infection. Several candidate drugs against the virus (viral inhibitors) or host targets (inhibitors of viral entry, enhancers of defensive reactivity) are currently in clinical trials for COVID-19. However, the constant emergence of new variants necessitates a constant reshuffling of specific preventive and therapeutic strategies. In addition, concerns about approved drugs, such as the potential toxicity and teratogenicity of molnupiravir (which may introduce mutations in the host) and the potential drug–drug interactions with Paxlovid^TM^, together with the potential induction of viral resistance to these drugs, call for new effective antivirals. Extensive studies with advanced technologies and methodologies are required to gain additional insight into the molecular mechanisms underlying host–virus interaction, virus mutation and evolution, and host immune and inflammatory responses as the bases for the development of novel therapeutic strategies.

The unprecedented global effort to investigate the host–pathogen interaction in COVID-19 has generated a massive amount of data that remains to be examined, understood and exploited to design optimal preventive and therapeutic strategies. Vaccination is the most economical and effective public health intervention for the prevention and control of infectious diseases in modern medicine. Up to now, more than 10 billion doses of anti-SARS-CoV-2 vaccines have been administered worldwide, including mRNA vaccines, adenovirus vaccines, and inactivated and subunit vaccines. While all vaccines offer some protection against SARS-CoV-2, tailor-made vaccines targeting specific variants or broadly protective antiviral vaccines have so far not been achieved. An open issue with all the anti-SARS-CoV-2 vaccines is the duration of the protection, which seems limited (at least when examining antibody titers). Thus, booster vaccination has been implemented worldwide with repeated injection (every six months) and with the use of different vaccines and adjuvants at each booster in order to broaden the capacity to recognize viral variants and prolong the duration of immune protection. New perspectives in vaccine development include the innovative concept of epigenomic adjuvanticity, i.e. an immunotherapeutic approach to reprogram innate responses towards better resistance and fewer side effects in response to future infections. Multi-omics studies can provide potentially valuable guidance for vaccine design and use, but these novel technologies require standardization and validation before they are sufficiently mature to effectively support clinical studies. We also stress here that the selection of experimental models in pre-clinical studies is critical because of the many important differences between humans and other animals that may substantially hamper clinical translatability.
